# Aging Systemic Milieu Impairs Outcome after Ischemic Stroke in Rats

**DOI:** 10.14336/AD.2017.0710

**Published:** 2017-10-01

**Authors:** Mengxiong Pan, Peng Wang, Chengcai Zheng, Hongxia Zhang, Siyang Lin, Bei Shao, Qichuan Zhuge, Kunlin Jin

**Affiliations:** ^1^Zhejiang Provincial Key Laboratory of Aging and Neurological Disorder Research, the First Affiliated Hospital, Wenzhou Medical University, Wenzhou, Zhejiang 325000, China; ^2^Institute for Healthy Aging, University of North Texas Health Science Center, Fort Worth, Texas 76107, USA

**Keywords:** ischemic stroke, systemic milieu, outcome, plasma, haptoglobin

## Abstract

Compelling evidence indicates that factors in the blood can profoundly reverse aging-related impairments, as exposure of aged mice to young blood rejuvenates adult stem cell function, improves cognition, and ameliorates cardiac hypertrophy. Systemic factors from mice can also extend the life span of a partner exposed to a lethal treatment or disease. These findings suggest that the systemic milieu of a healthy young partner may be beneficial for an aged organism. However, it is unknown whether a healthy young systemic milieu can improve functional recovery after ischemic stroke. Intraperitoneal administration of young plasma into aged rats after ischemic stroke induced by distal middle cerebral artery occlusion (dMCAO) reduced infarct volume and motor impairment, compared with vehicle group. On the contrary, intraperitoneal administration of plasma from aged rats into young ischemic rats worsened brain injury and motor deficits. Using a proteomic approach, we found that haptoglobin levels were significantly increased in serum of aged rats and that intraperitoneal administration of haptoglobin impaired outcome after ischemic stroke in young rats. Our data suggest that the aging systemic milieu plays a critical role in functional outcome after ischemic stroke.

Aging is associated with a striking increase in the incidence of ischemic stroke, which is a leading cause of disability among those age 70 years and older [1]. In addition, both preclinical and clinical stroke studies show that age correlates with poor histologic outcome and worsened neurobehavioral deficits after ischemic stroke. The underlying mechanism remains elusive, but recent studies showing a therapeutic effect of the infusion of blood from young animals into old animals provide a possible link [2, 3].

Systemic factors in the blood can profoundly reverse aging related impairments. The age-related decline of muscle progenitor cell activity can be restored, and damaged muscle repaired after exposure to a "youthful’’ systemic milieu through heterochronic parabiosis, an approach for exchanging systemic milieu of two organisms. On the contrary, exposing a young mouse to an old systemic milieu can inhibit myogenesis [4, 5]. Age-related cardiac hypertrophy can also be reversed by exposure to a young systemic milieu, accompanied by reduced cardiomyocyte size and molecular remodeling [3]. Similarly, systemic administration of young plasma to aged mice increased neurogenesis and improved age-related cognitive impairments [2, 6], while exposing a young mouse to an old systemic milieu or to plasma from old mice impaired cognitive function [6]. These observations suggest that cognitive impairments during aging may be attributed in part to changes in blood-borne systemic factors [2, 6]. A recent study also documented that a healthy mouse could extend the life span of a partner exposed to a lethal treatment or disease through heterochronic parabiosis [7]. Taken together, these findings indicate that signals from the systemic milieu are closely associated with the brain dysfunction, and raise questions about the role of systemic factors in worsened functional recovery after ischemic stroke and the therapeutic potential of blood-borne factors from young organisms.

In this study, we demonstrate that intraperitoneal administration of young plasma into aged ischemic rats significantly improved functional outcome. On the contrary, intraperitoneal administration of plasma from aged rats into young ischemic rats worsened brain injury and motor deficits. The poorer functional outcome elicited by exposure to old plasma may be mediated, at least in part, by increased haptoglobin in the aged blood. Our data suggest that the aging systemic milieu plays a critical role in functional recovery after ischemic stroke, and elucidates a previously unrecognized role for haptoglobin in the prognosis of ischemic stroke.

**Figure 1.**
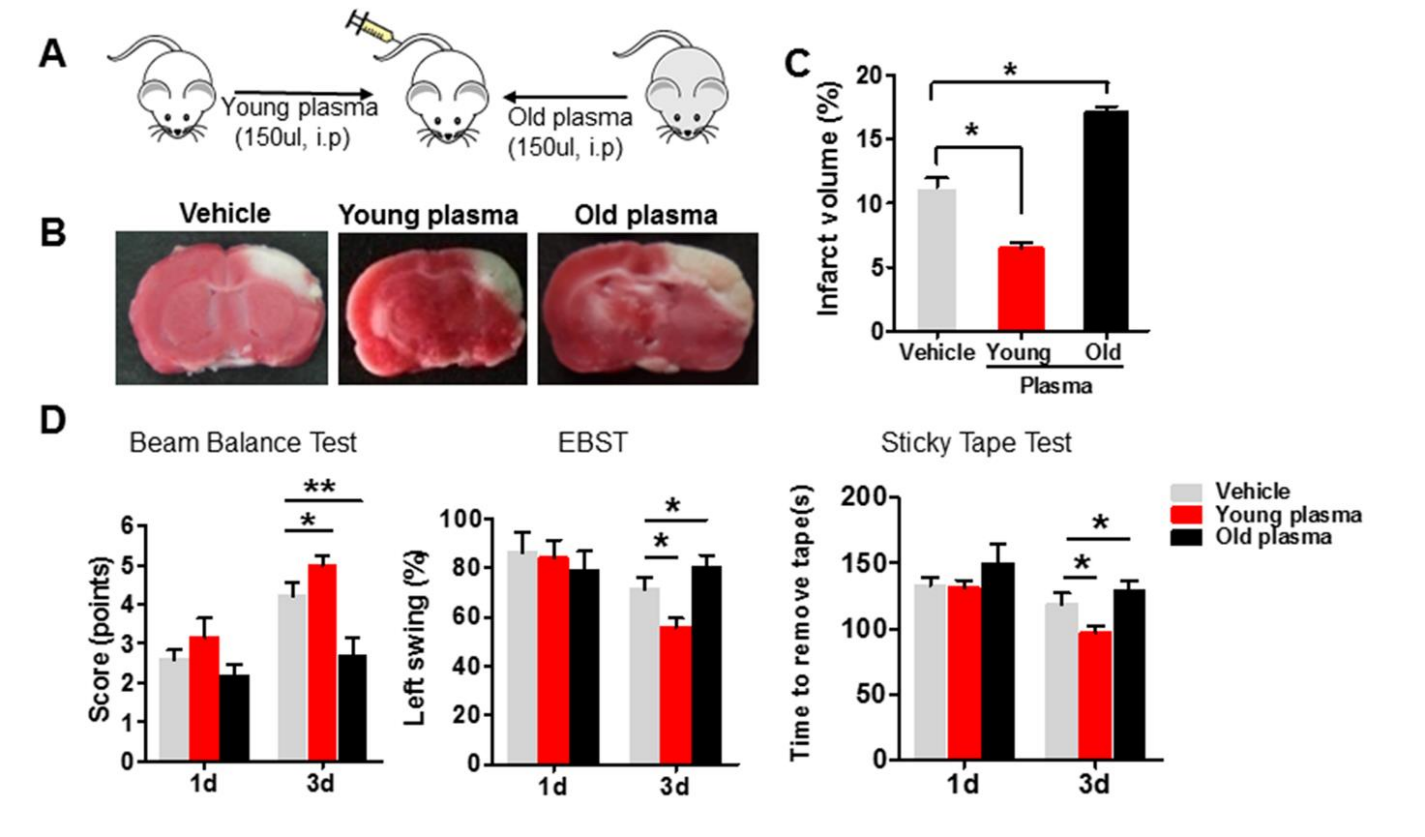
Administration of old plasma into young ischemic rats worsensyoungyoungwrosew were outcomeafter ischemic stroke **(A)** Schematic illustrating intraperitonealinjection of young (2-month-old) or old (22-month-old) plasma into young ischemic rats 1 hr after onset of ischemia, twice per day for 3 days. **(B)** Infarct areas (white) in TTC-(red) stained coronal brain section from vehicle, young and old plasma-treated rats at 3 days after stroke. **(C)** Quantitative analysis of infarct volume in vehicle, young and old plasma-treated rats at 3 days after ischemic stroke. **(D)** Motor function deficits of young ischemic rats were assessed by beam balance test, EBST and sticky tape test on the 1^st^ and 3^rd^ day after administration with vehicle, young, or old rat plasma. Values presented as mean ± SEM. *P<0.05; **P<0.01, compared with vehicle-treated group. N=7-12 per group.

## MATERIALS AND METHODS

### Focal cerebral ischemia

Young (2-month-old) and aged (20-22-month-old) male Sprague-Dawley rats from the Animal Center of Shanghai Branch, Chinese Academy of Sciences and aged (24-month-old) male Fisher 344 rats from the National Institute on Aging aged rodent colony were anesthetized with 2.0% isoflurane in 70% N_2_O/30% O_2_. Permanent distal middle cerebral artery occlusion (dMCAO) was performed as described previously [8, 9]. Briefly, a 2-cm incision was made under the surgical microscope between the right orbit and tragus, the temporal muscle was retracted laterally, and a 3-mm diameter craniotomy was made just rostral to the foramen oval. A small burr hole (2-mm) was made with a high-speed microdrill through the outer surface of the semitranslucent skull just over the visible middle cerebral artery (MCA) at the level of the inferior cerebral vein. The inner layer of the skull was removed with fine forceps, the dura and arachnoid were opened, and right MCA occlusion (MCAO) was performed by electrocoagulation with a small-vessel cauterizer without damaging the brain surface. If the brain surface was visibly damaged or if the MCA bled owing to incomplete artery occlusion/coagulation, the animal was euthanized and not used for experiments. The temporal muscle was repositioned and the skin was closed. After surgery, rats were placed in a cage under an infrared heating lamp until they recovered from anesthesia. Rectal temperature was maintained at 37.0 ± 0.5°C using a thermostat-controlled heating pad (Harvard Apparatus, Holliston, MA, USA). All animal procedures were approved by the Institutional Animal Care and Use Committees of Wenzhou Medical University and University of North Texas Health Science Center, and conducted according to the National Institutes of Health (NIH) Guide for the Care and Use of Laboratory Animals. Every effort was made to minimize suffering and to reduce the number of animals used.

**Figure 2.**
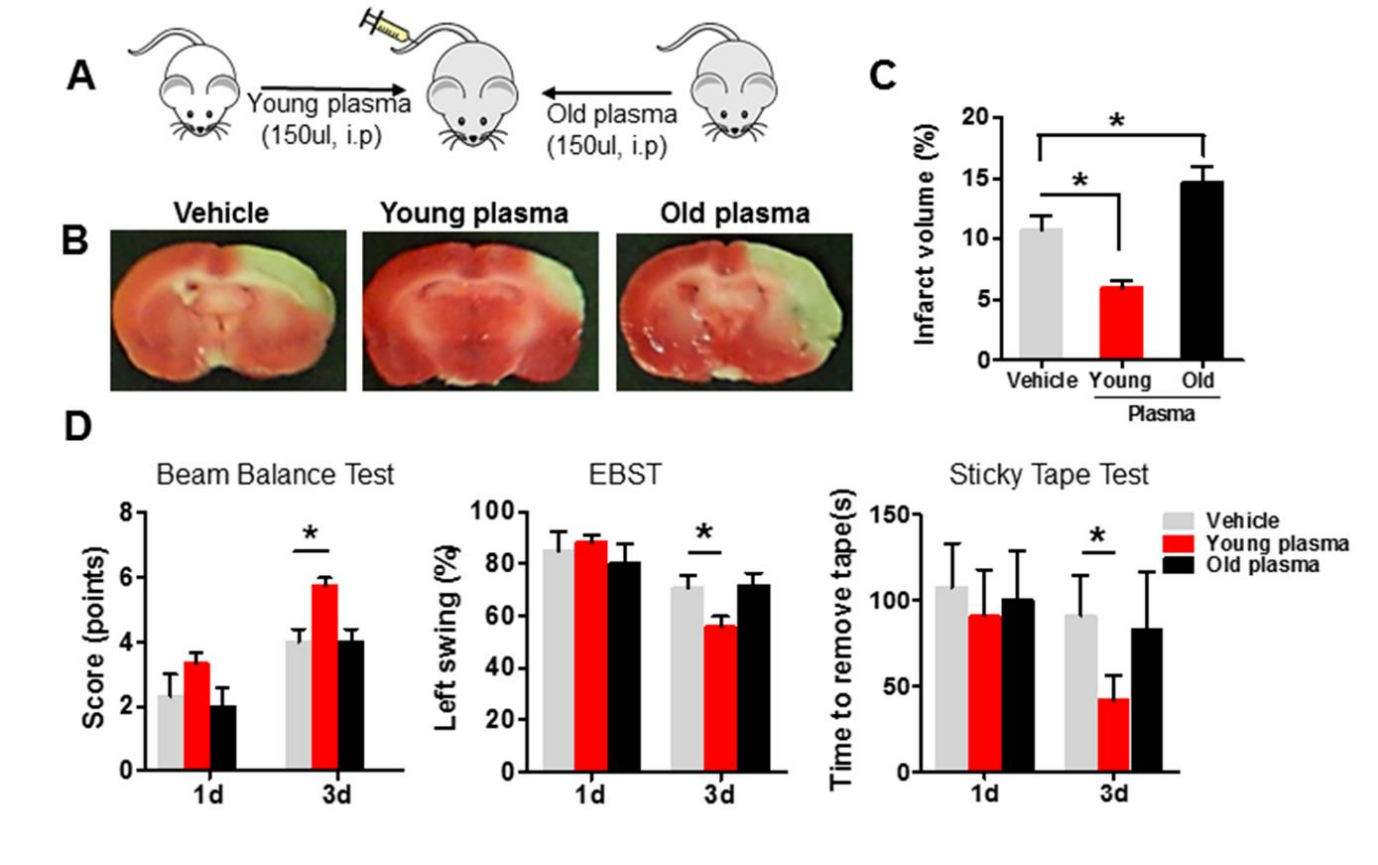
Young plasma reduces youngyounginfarct volume and improves neurobehavioral deficits in aged ischemic rats after stroke **(A)** Schematic illustrating intraperitonealinjection of young (2-month-old) or old (22-month-old) plasma into aged ischemic rats 1 hr after onset of ischemia. **(B)** Representative images of TTC staining in cerebral sections of vehicle, young and old plasma-treated group, twice per day for 3 days after stroke. **(C)** Infarct volume (expressed as percentage whole brain volume) in vehicle, young and old plasma-treated aged rats 3 days after stroke. **(D)** Neurobehavioral tests includingbeam balance test, EBST and sticky tape test were performedon the 1^st^ and 3^rd^ day after injection with vehicle, young, or old rat plasma into aged rats. Values presented as mean ± SEM. *P<0.05, compared with vehicle-treated group. N=7-12 per group.

### Infarct volume measurement

Brains were removed 3 or 15 days after ischemia and 2-mm coronal sections were stained with 2,3,5-triphenyltetrazolium hydrochloride (TTC) [10]. In some cases, frozen sections were also cut and stained with hematoxylin and eosin (H&E) [11]. Infarct area and total brain area were measured by a blinded observer using the NIH Image program, and areas were multiplied by the distance between sections to obtain the respective volumes. Infarct volume was calculated as a percentage of the volume of the whole brain, as described previously [12].

### Plasma and haptoglobin administration

Fresh blood was obtained from healthy young (2-month-old) and aged (20-22-month-old) male Sprague-Dawley rats (n=7 per group), and then collected into sterile tubes containing ethylenediaminetetraacetic acid (EDTA). Samples were centrifuged at 3000 rpm for 15 min and plasma was taken and aliquots stored at -80 °C until further use. Pooled plasma from 5 rats or haptoglobin (Abcam; 0.5g/L) was administered to recipient rats in a volume of 150 μL by the intraperitoneal route twice per day for 3 or 15 days. Informed consent was obtained from the human subjects, and all protocols were approved by the Medical Ethics Committee of the First Affiliated Hospital of Wenzhou Medical University.

### 2D-DIGE and mass spectrometry analysis

Two-dimensional difference gel electrophoresis (2D-DIGE) was used to ascertain the proteomic profiles of young and old rat plasma as well as healthy young (18 ± 3.6 years old), middle-aged (36 ± 5.7 years old) and aged (79 ± 4.3 years old) human subjects (n=10 per group) according to the standard Applied Biomics protocol (Applied Biomics, Hayward, CA). For 2D-DIGE analysis, plasma protein was labeled separately with Cy3 and Cy5 and simultaneously separated on a single 2D gel using isoelectric focusing in the first dimension (pH gradient 3-10 L) and SDS-PAGE (10% acrylamide) in the second dimension. After electrophoresis, fluorescent-labeled proteins in the 2D gels were scanned using a Typhoon variable mode imager (Applied Biomics, Hayward, CA). DeCyder 2D software (version 6.5) was used to calculate the spot ratio. Proteins of interest were automatically picked out from the 2D gel with Ettan Spot Picker and then identified using a matrix-assisted laser desorption/ionization (MALDI)-time-of-flight/time-of-flight (ToF/ToF) mass spectrometer (Applied Biomics, Hayward, CA).

### Enzyme-linked immunosorbent assay

Rat and human haptoglobin enzyme-linked immunosorbent assay (ELISA) was used to determine the concentration of haptoglobin in rat and human plasma according to the manufacturer’s instructions (TSZ Biological Trade Co., Waltham, MA, and R&D Systems, Minneapolis, MN, USA).

### Western Blotting

Western blotting was conducted as previously described [13]. Plasma protein (30 µg) was boiled at 100°C in the SDS sample buffer for 5 min, electrophoresed on 12% SDS-PAGE gels, and transferred into poly-vinyldifluoridine membranes, which were incubated overnight at 4°C using one of the following primary antibodies: (1) rabbit anti-rat haptoglobin (1: 10,000; Abcam), (2) mouse anti-human haptoglobin α (1: 2,000; Santa Cruz Biotechnology) and (3) chicken anti-albumin (1: 1,000; Abcam). Membranes were washed with 0.1% Tween-20 in 1xPBS, incubated at room temperature for 60 min with horseradish peroxidase conjugated anti-mouse, anti-rabbit, or anti-chicken secondary antibody (Santa Cruz Biotechnology; 1: 3,000), and washed three times for 15 min with 0.1% Tween-20 in 1 x PBS. Peroxidase activity was visualized by chemiluminescence (NEN Life Science Products, Boston, MA, USA). Densitometry measurements were normalized to albumin. Differences in protein expression were quantified using a GS-710 calibrated imaging densitometer and QUANTITY ONE software (Bio-Rad).

### Neurobehavioral tests

Rats underwent the neurobehavioral tests described below to evaluate functional outcome 1, 3, 7, and 15 days after ischemia. All were trained 3 days before induction of ischemia. Observers were blinded to the experimental condition.

#### Beam balance test

The beam balance test was used to assess deficits in coordination and integration of motor movement, especially in the hindlimb [14], and performed as previously described with modification [15, 16]. Rats were placed on the middle of a square wooden bar (2.5 x 2.5× 122 cm) at a height of 42 cm and scored as follows: rat unable to stay on the beam, 0 points; rat unable to move, but able to stay on the beam, 1 points; rat tries to turn to left or right side of the beam, 2 points; rat turns to left or right side and walks on the beam with more than 50% foot-slips of the affected hindlimb, 3 points; rat traverses the beam with more than one foot-slip, but less than 50%, 4 points; rat has only one slip of the hind limb, 5 points; rat traverses the beam without any slips of the hind limb, 6 points. Rats were placed on the beam three times and the mean score of three trials was used.

**Figure 3.**
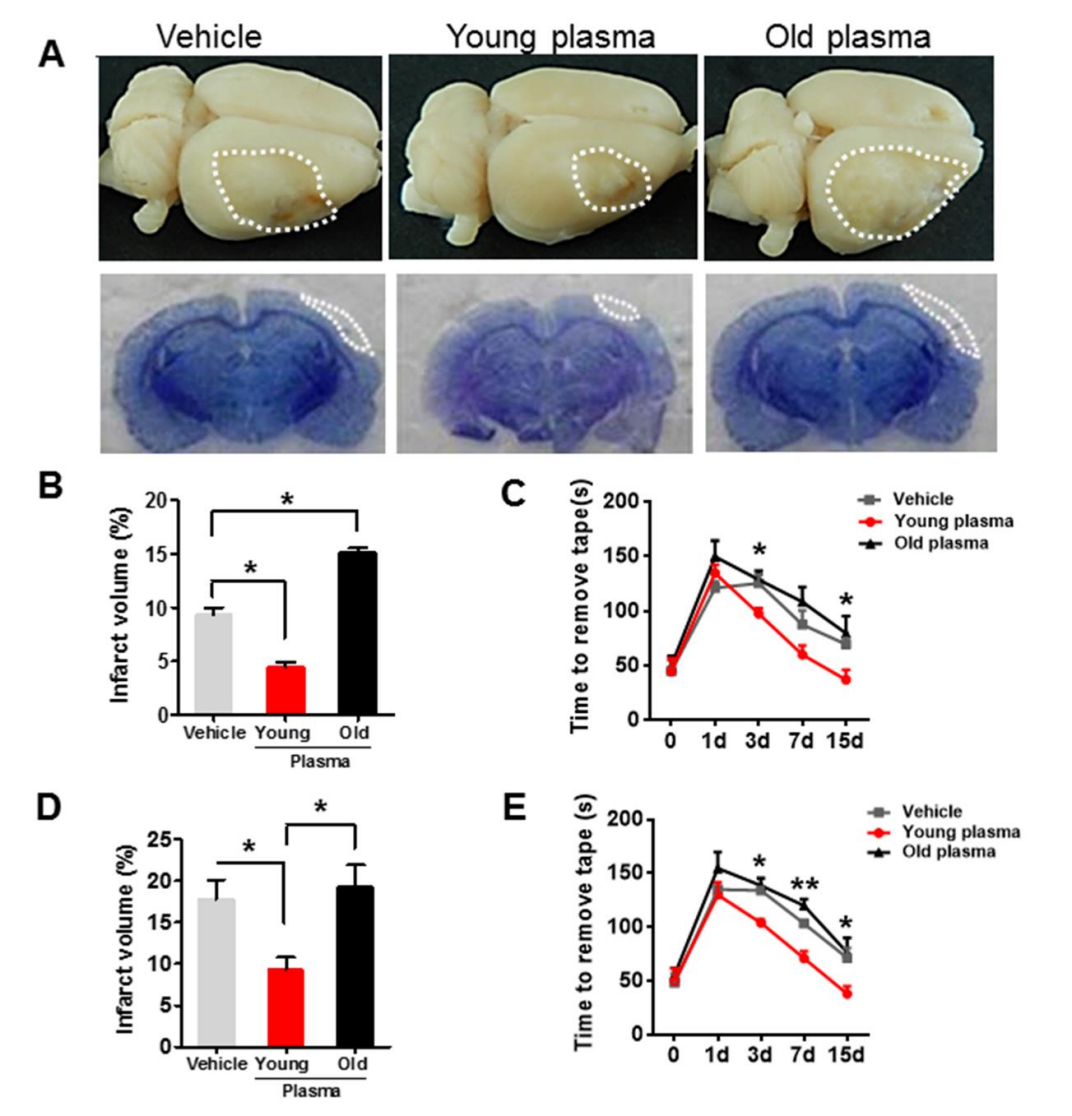
Old plasma administration inhibits long-term recovery after experimental stroke in young ischemic rats **(A)** Representative images of lesion volume at low magnification view (top panel) and H&E-stained coronal brain sections (bottom panel) in vehicle, young plasma and old plasma-treated young rats 15 days after stroke. **(B)** Quantitative analysis of infarct volume in vehicle, young and old plasma-treated young rats 15 days after ischemic stroke. **(C)** Sticky tape test scores in young rats at 1, 3, 7 and 15 days after treatment with vehicle, young plasma and old plasma. **(D)** Quantitative analysis of infarct volume in vehicle, young and old plasma-treated aged rats 15 days after ischemic stroke. **(E)** Sticky tape test scores in aged rats at 1, 3, 7 and 15 days after treatment with vehicle, young plasma and old plasma. Values presented as mean ± SEM. *P < 0.05; **P < 0.01, compared with vehicle-treated group. N=7-12 per group.

#### Elevated body swing test (EBST)

The elevated body swing test was used to test asymmetric motor behavior, was first described by Hoyman *et al* [17]. Rats held by the base of the tail were raised ≈10 cm above the testing surface. The initial direction of body swing, constituting a turn of the upper body of >10° to either side, was recorded in three sets of ten trials, performed over 5 min. The number of turns in each (left or right) direction was recorded, and the percentage of turns made to the side contralateral to the ischemic hemisphere (percent left-biased swing) was calculated., Average scores were determined for each rat.

**Figure 4.**
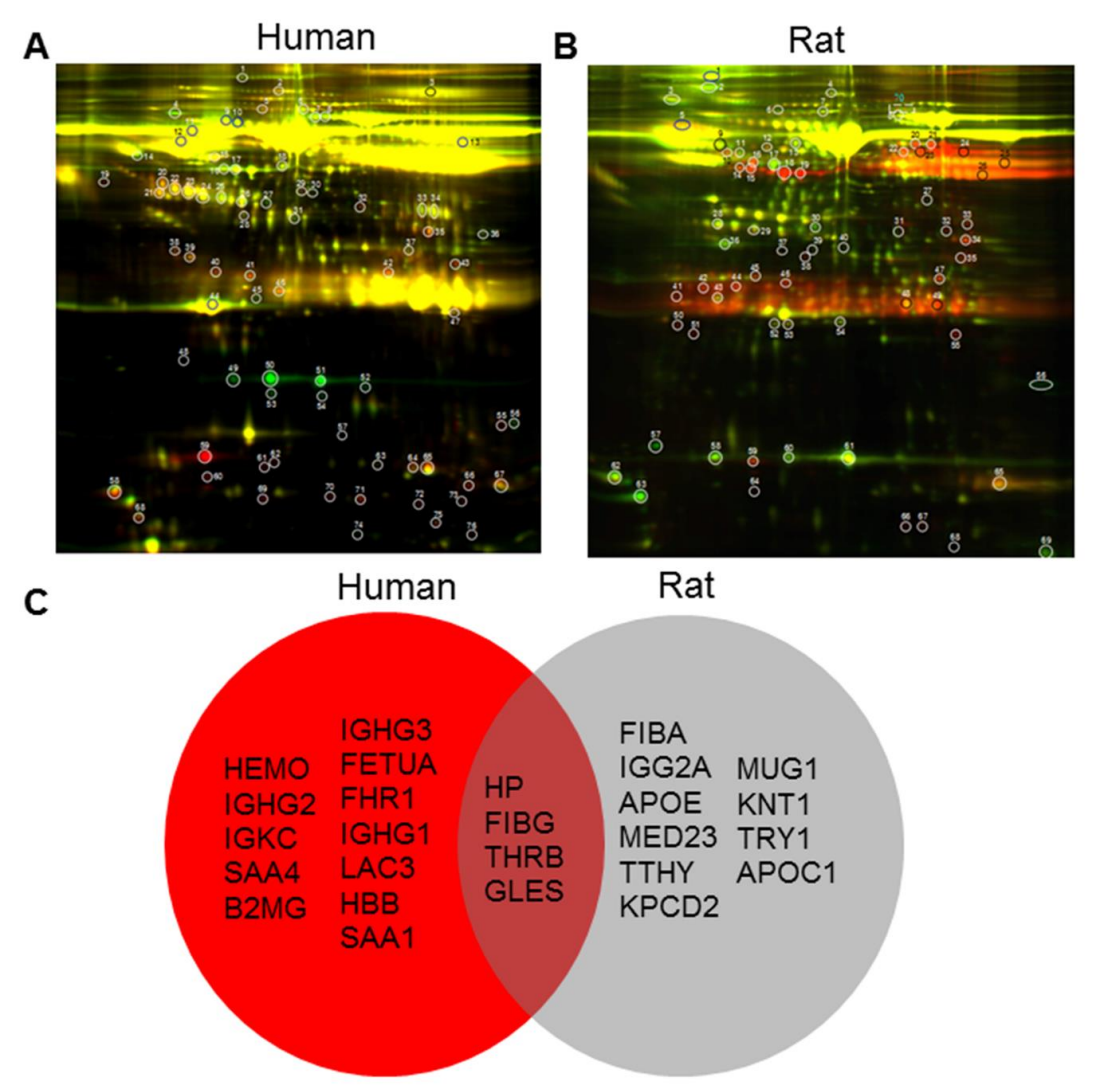
Differentially expressed proteins identified from the 2D-DIGE profiling of young and old plasma of human and rat **(A)** Protein from rat young plasma was labeled with Cy3 (green), protein from rat old plasma was labeled with Cy5 (red), and samples were mixed prior to 2-dimensional PAGE (horizontal axis, pI; vertical axis, M_r_). **(B)** Cy3 (green) was used to label young human plasma and Cy5 was used to label old human plasma. N=10/group. **(C)** Venn diagram of differentially expressed protein spots identified from the 2D-DIGE profiling of young and old human (red) and rat plasma (grey). In the brown intersection are shown four factors changed in both proteomic screens.

#### Sticky tape test

The test analyzes forepaw sensory function, including presence of neglect, as well as motor impairments of the forepaw [18], and was performed as previously described [19]. Briefly, a 1.5-cm wide tape was attached to each rat paw. The tape was attached to the contralateral forepaw of the ischemic hemisphere. The time to remove the tape from each paw was recorded. Five trials were performed and the median value was taken for each rat.

#### Corner test

The corner test [20] was used to test sensorimotor and postural asymmetry [21, 22]. The rat was placed between cardboards, each with a dimension of 30 x 20 x 1 cm^3^. The two boards were gradually moved to enclose the rat from both sides and encourage the rat to enter a 30º corner with a small opening along the joint between the two boards. When the rat entered deep into the corner, the vibrissae on both sides were contacted by the boards. The rat reared forward and upward, then turned back to face the open end. Twenty trials were performed for each rat. Lateral Index (LI) was calculated as the difference between right and left turns divided by the total number of tests. Then testing value was divided by the training value for calibration.

### Statistical analysis

Quantitative data were expressed as mean ± standard error of the mean (SEM) from the indicated number of experiments. The statistical significance of differences between means was analyzed by one-way analysis of variance (ANOVA) followed by Fisher PLSD *post hoc* tests. Behavioral data were analyzed by two-way ANOVA with repeated measures, followed by *post hoc* multiple comparison tests (Fisher PLSD or Student’s paired *t* test with Bonferroni correction). *P* values < 0.05 were considered significant.

## RESULTS

We first investigated whether the young and old systemic milieu differentially impacted out come in young rats subjected to dMCAO. Young or old plasma was intraperitoneally injected into young rats1 hr after the onset of ischemia (Fig. 1A) and histologic outcome was assessed by measuring infarct size, determined by TTC staining of coronal brain slices [10] (Fig. 1B). Infarct volume was decreased 3 days after young plasma was administered, compared with the saline-treated group. In contrast, rats treated with old plasma showed an extension of infarct area (Fig. 1B) and increased infarct volume, compared with that observed after vehicle or young plasma treatment (Fig. 1C).

Consistent with histologic results, post-ischemic functional deficits, measured using the beam balance test, sticky tape test and EBST (Fig. 1D), were significantly improved in young rats 3 days after treatment with young plasma, compared with vehicle treated rats. Conversely, neurological deficits were greater in old plasma- than in saline- or young plasma-treated young rats.

Then we asked whether the young and old systemic milieu also affected outcome in aged ischemic rats (Fig. 2A). As observed for young rats, intraperitoneal administration of young plasma into aged ischemic rats reduced infarct volume (Fig. 2B and C) and ameliorated motor impairment 3 days after focal ischemia (Fig. 2D), compared with the vehicle-treated group. On the contrary, intraperitonea*l* administration of old plasma into aged ischemic rats worsened their brain injury, but motor deficits were not significantly affected, 3 days after treatment, compared with young rats (Fig. 2B, C and D).

Next, we examined the effect of repeated administration of young or old plasma on long-term recovery in young and aged ischemic rats. Plasma pooled from 5 young or 5 aged rats, or vehicle, was injected into rats, twice per day for 15 days, after dMCAO and neurobehavioral test was performed at 1, 3, 7 and 15 days. Histologic outcome was determined by H&E staining 15 days after treatment, which showed cavitary cerebrocortical lesions within the distal MCA territory (Fig. 3A). Compared with saline-treated controls, young rats treated with young plasma exhibited reduced cerebral lesion volumes and improved neurological deficits (Fig. 3B and C), whereas treating young rats with old plasma had the opposite effects (Fig. 3B and C). Infarct size after 15 days was larger in aged than in young vehicle-treated rats (Fig. 3B and D), but the differential effects of young vs. old plasma on infract size and functional deficits were again observed (Fig. 3D and E).

**Figure 5.**
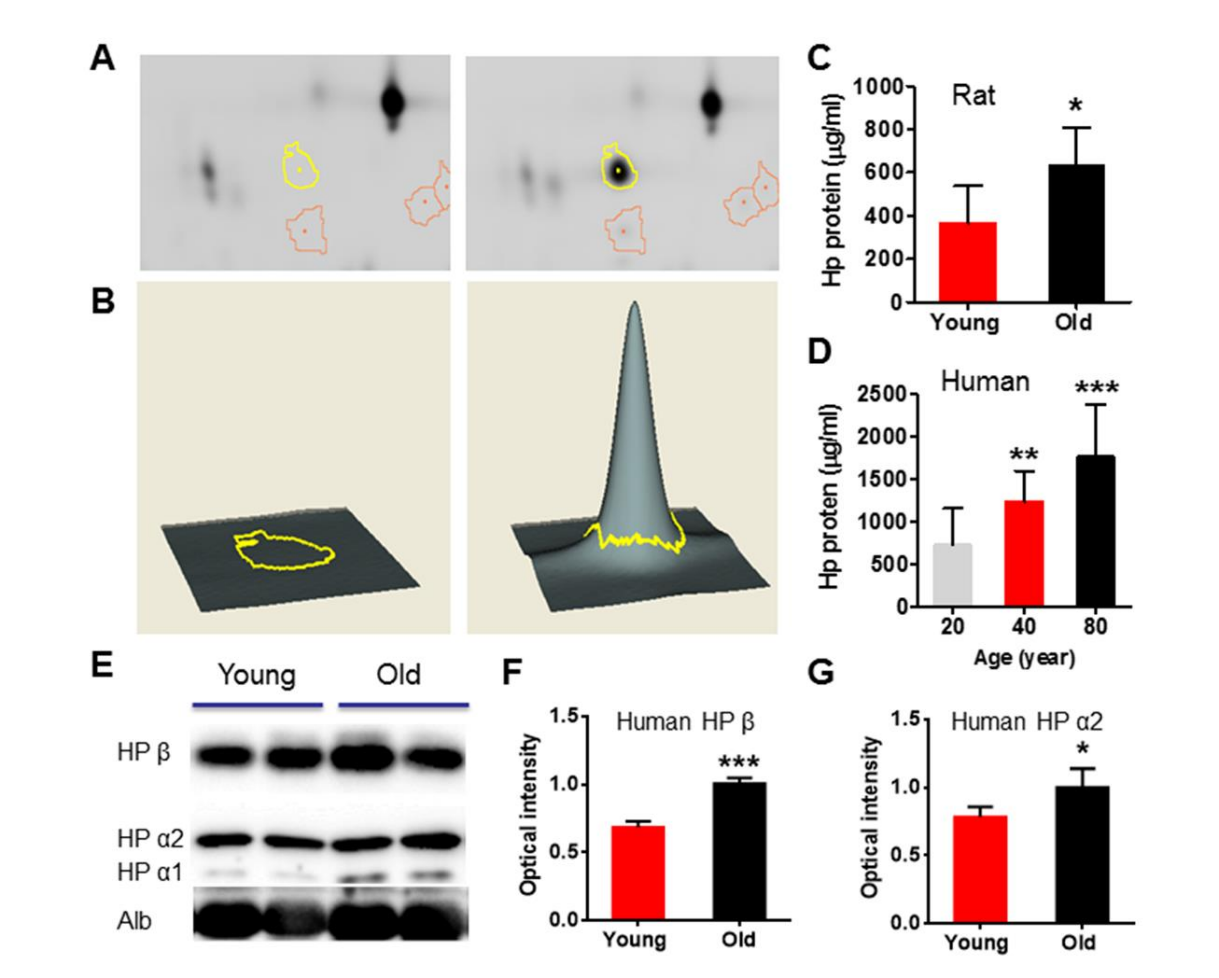
Circulation level of haptoglobin is significantly increased in both old rat and human plasma **(A)** DeCyder Analysis shows the location of a 2D-DIGE gel with yellow line pointing to protein spots, identified as haptoglobin, that was differentially expressed across age, >72.5 change in abundance. **(B)** 3-D view simulation of a close-up of the region of 2D-DIGE gel image, and the associated graph of representative up-regulated haptoglobin during aging. **(C)** ELISA shows that haptoglobin was increased in young and old rat plasma. N=7 rats per group. **(D)** ELISA shows that haptoglobin concentration in the plasma of 20-, 40-, and 80-years-old human subjects. **(E)** Western blot analysis confirms that increased level of haptoglobin α1, α2 and β in old human plasma. (**F** and **G**) Relative optical intensity of haptoglobin β and α2. Data were from 10 per group. Values were presented as mean ± SEM. *P < 0.05, **P < 0.01, ***P < 0.001.

These data suggest that differences between systemic factors in young and aged rats may underlie differences in functional outcome. To identify candidate factors that could contribute to such differences, we used 2D-DIGE and mass spectrometry to compare the plasma profiles from young and aged rats as well as healthy young, middle-aged and aged human subjects. We used *DeCyder* 2-D Differential Analysis Software to analyze the difference between plasma proteomes. About 2267 protein spots in human (Fig. 4A) and 2147 protein spots in rats (Fig. 4B) were detected. Up to 778 (34.3%) protein spots in human plasma samples and about 1080 (50.3%) proteins in rat plasma were found to be significantly different in abundance across subjects ages. Among these, 422 protein spots were increased and 356 protein spots were decreased in young compared to aged human plasma, and 345 protein spots were increased and 735 protein spots were decreased in young compared to aged rat plasma. We selected 20 spots from human and rat groups for protein identification, and found 16 factors significantly different between young and old human plasma, and 14 factors altered between young and old rat plasma (Fig. 4C). Interestingly, only four factors - haptoglobin (HP), fibrinogen gamma chain (FIBG), gelsolin (GELS) and prothrombin (THRB) were changed in plasma of both human and rats during normal aging (Fig. 4C). Haptoglobin has three common phenotypes (Hp1-1, Hp2-1 and Hp2-2) in humans, and binds free hemoglobin released from erythrocytes with high affinity. Haptoglobin levels in rat brain increase with aging [23] and haptoglobin polymorphisms or phenotypes have been associated with differences in the incidence and severity of vascular diseases, including stroke [24-26]. We confirmed that haptoglobin was increased in the plasma of aged rats and human subjects by ELISA and Western blots (Fig. 5).

**Figure 6.**
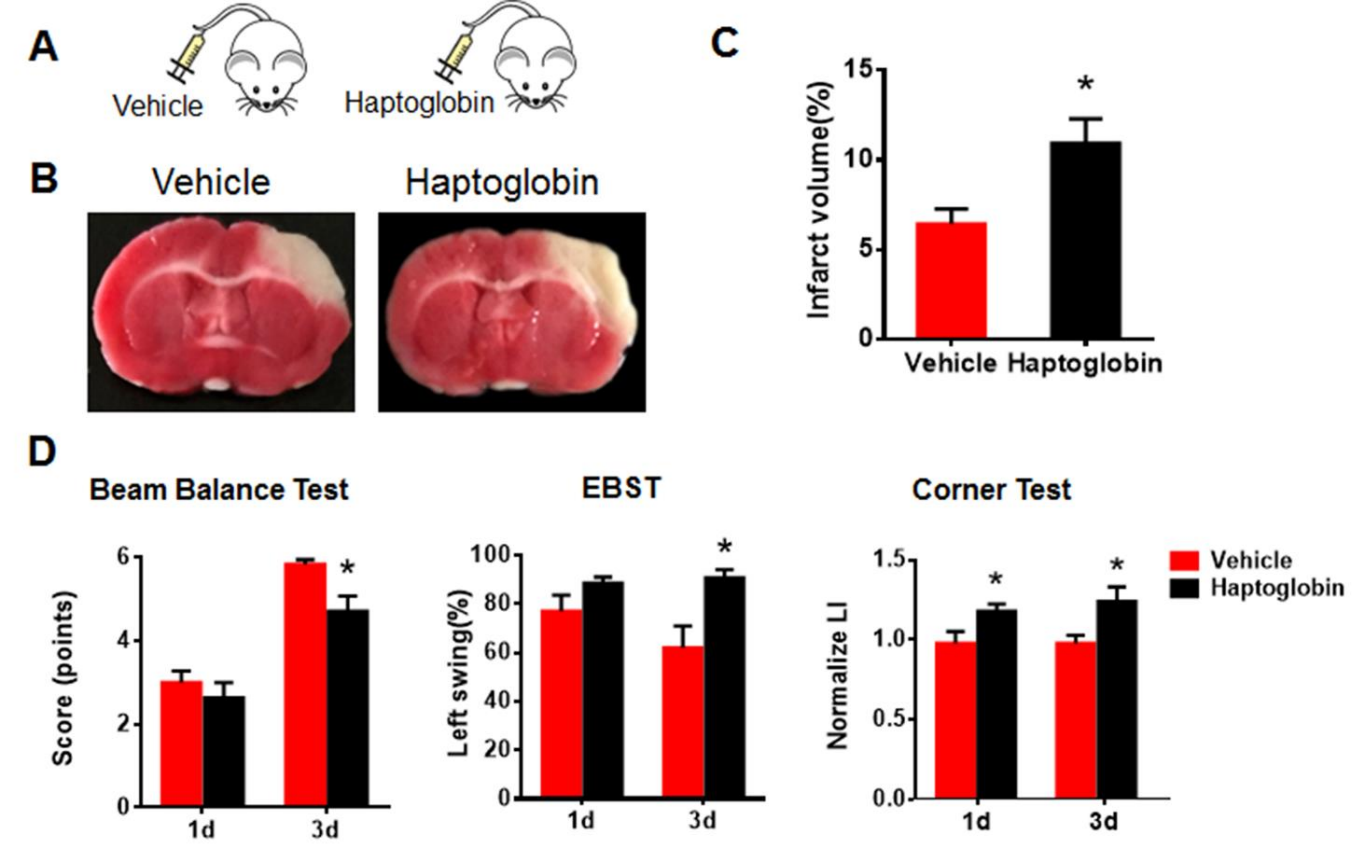
Systemic exposure to haptoglobin increases infarct volume and deteriorates neurobehavioral deficits in young ischemic rats **(A)** Schematic of young rats injected intraperitoneally with haptoglobin (0.5 g/L in 150 µl) or vehicle 1 hr after ischemic stroke. **(B)** Representative images of TTC staining in coronal brain sections of vehicle- or haptoglobin-treated young ischemic rats. **(C)** Quantitative analysis of infarct volume in vehicle- and haptoglobin-treated young rats 3 days after ischemic stroke. **(C)** Neurobehavioral deficitswere determined by beam balance test, EBST and corner test in young ischemic rats after injection with vehicle or haptoglobin. Values presented as mean ± SEM. *P< 0.05, **P< 0.01. N=7-12 per group.

**Figure 7.**
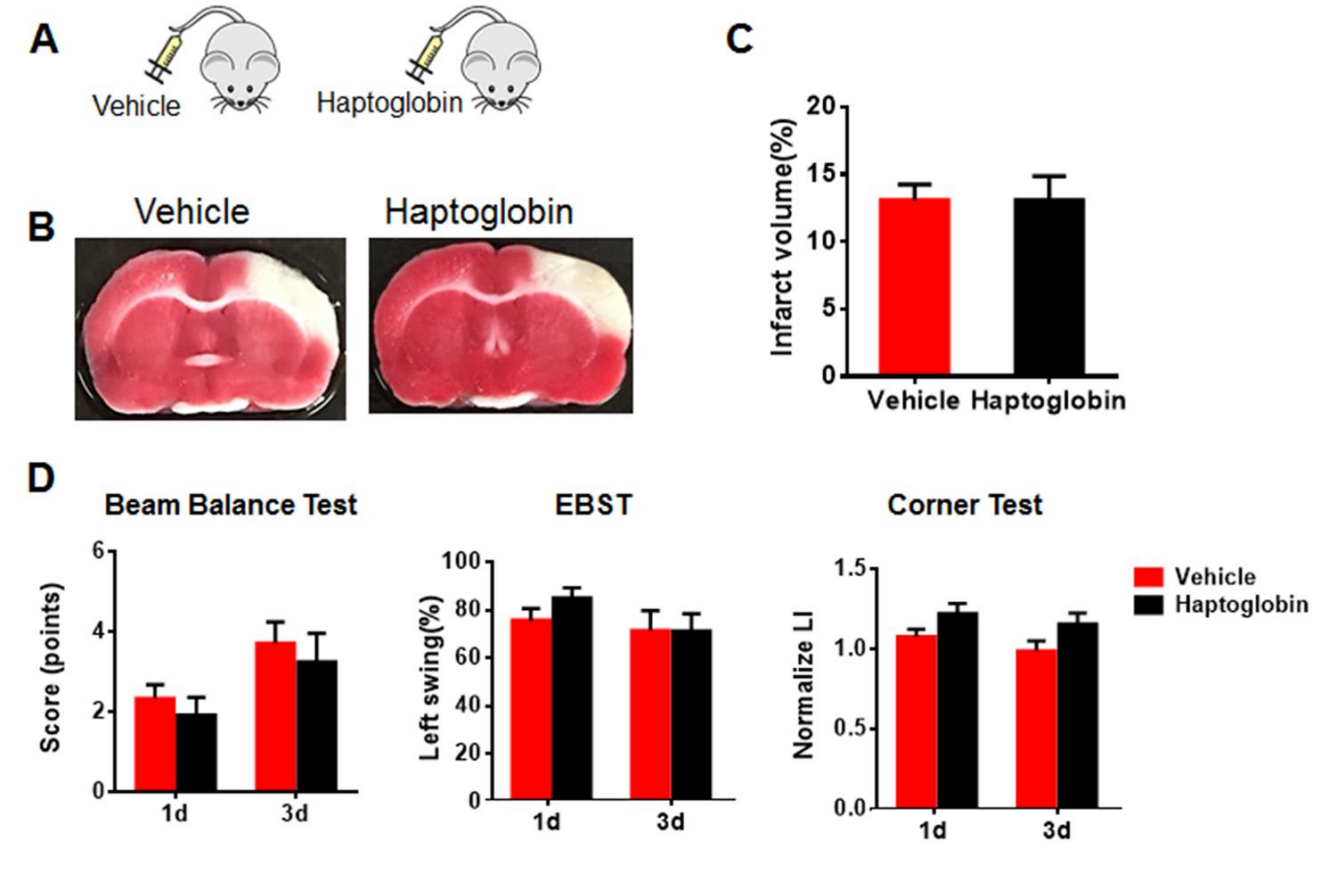
Systemic exposure to haptoglobin affect infarct volume and neurobehavioral deficits in aged ischemic rats **(A)** Schematic of aged rats injected intraperitoneally with haptoglobin (0.5 g/L in 150 µl) or vehicle 1 hr after ischemic stroke. **(B)** Representative images of TTC staining in coronal brain sections of vehicle- or haptoglobin-treated aged ischemic rats. **(C)** Quantitative analysis of infarct volume in vehicle- and haptoglobin-treated aged rats 3 days after ischemic stroke. **(C)** Neurobehavioral deficits were determined by beam balance test, EBST and corner test in aged ischemic rats after injection with vehicle or haptoglobin. Values presented as mean ± SEM. N=8-14 per group.

Since haptoglobin is as an age-related systemic factor and a significant risk factor for acute myocardial infarction, stroke, and heart failure [24], we investigated its possible role in functional recovery in our ischemic stroke model. We injected haptoglobin (0.5 g/L in 150 µl intraperitoneally) into young and aged rats twice per day for 3 days after dMCAO [27]. Neurobehavioral tests were performed on days 1 and 3 and infarct volume was determined after the last injection (Fig. 6A). In young rats given haptoglobin, infarct volume was greater (Fig. 6B and C), and neurological deficits were worse at 1 and 3 days (corner test) and 3 days (beam balance test and EBST) (Fig. 6D). However, haptoglobin had little effect in aged ischemic rats (Fig. 7).

## DISCUSSION

Here, we found that systemic administration of young plasma into an aged ischemic rat reduced infarct volume and improved neurobehavioral deficit, while exposing a young ischemic rat to plasma from aged rats had the opposite effects. In addition, we confirmed that haptoglobin, an acidic glycoprotein present in most body fluids of humans and other mammals, was increased in blood of both old rats and aged human subjects, and reproduced the effect of old plasma in worsening outcome in young ischemic rats. Our data indicate that the systemic milieu plays a critical role in functional recovery after experimental ischemic stroke. This suggests that interventions to minimize the effects of detrimental (aging-related) factors or provide beneficial (youth-related) factors from the milieu might improve outcome of stroke patients in clinic.

Indeed, clinical studies have documented that the prognosis of ischemic stroke in the young is much better than in the elderly, with lower mortality, less frequent recurrence and better functional recovery [28, 29]. Although the underlying mechanisms remain unclear, increasing age is a strong predictor of poor outcome, even within the 15- to 45-year age range [30]. Recent studies have shown that young blood contains factors that can rejuvenate stem/progenitor cells in aged tissues, including muscle [31], liver [4], heart [3] and the brain [6], and ameliorate cardiac hypertrophy [3]. In addition, young blood can counteract aging and enhances cognitive processes [2]. Our data confirm and extend previous findings that signals from the aging systemic milieu are closely associated not only with brain dysfunction, but also with the prognosis in ischemic stroke. The therapeutic effect of the infusion of blood from young into old animals has fundamental implications for strategies to prevent or treat ischemic stroke are not only focused within the central nervous system (CNS), but also systemic milieu.

To identify the factors in the blood that might modify stroke outcome, we analyzed the protein profiles of young and old blood using a proteomic approach, and identified factors with levels that change with age. Haptoglobin emerged as a strong candidate as it was increased in blood of both old rats and aged human subjects, consistent with previous studies that levels of haptoglobin in blood [32, 33], liver, cerebrospinal fluid (CSF) [23] and hippocampus [23] increase with aging in human and rodents. Haptoglobin is likely to play an important role in suppressing inflammatory responses, as it binds free hemoglobin released from ruptured red cells, and its plasma level increases in response to inflammatory stimuli such as infection and autoimmune reaction [34]. In addition, haptoglobin levels increase in various pathological conditions both in human and animals, and may be a sensitive marker of blood-brain barrier dysfunction [35]. Some studies show increased levels of haptoglobin was found in CSF from patients with Alzheimer’s disease (AD) [36], Parkinson’ disease (PD) and Huntington’s disease (HD) [37, 38]. Although the role of haptoglobin in these diseases is not yet fully understood, haptoglobin may be involved in pathogenesis or serve as a diagnostic marker. For example, elevated haptoglobin is associated with increased risk for acute myocardial infarction, stroke, and heart failure [24]. In this study, we found that the poorer functional outcome elicited by exposure to old plasma may be mediated, at least in part, by increased haptoglobin in the aged blood, which has implications for understanding the molecular mechanism that links age-related differences in disease prognosis and age-related changes in the systemic milieu. Loffredo et al [2] identified a "youthful" systemic factor, growth differentiation factor 11, that can reverse cardiac hypertrophy in old mice, and Villeda et al[2] found that blood-borne factors in young blood can reverse age-related cognitive defects and impairment of synaptic plasticity in mice. Villeda *et al* [6] also discovered that levels of C-C motif chemokine 11 (CCL11) are elevated in plasma and cerebrospinal fluid of aging humans, and that increasing peripheral CCL11 levels in young mice impaired adult neurogenesis, learning and memory.

In addition to haptoglobin, our DIGE/MS study identified several additional proteins that show differential expression in plasma from rats and humans of different ages. Although we have not characterized their effects in this study, these factors may also be candidates for a role in functional recovery after ischemic stroke. They include β2-microglobulin (B2M), a component of major histocompatibility complex class 1 molecules, which has been shown previously to be increased in blood of aging human and rats [6].
